# Tetraspanin-based immunocapture for high-depth proteomic profiling of extracellular vesicles from cerebrospinal fluid for biomarker discovery

**DOI:** 10.1186/s12014-025-09579-9

**Published:** 2026-01-17

**Authors:** Elizabeth R. Dellar, Iolanda Vendrell, Roman Fischer, Alexander G. Thompson

**Affiliations:** 1 Nuffield Department of Clinical Neurosciences, University of Oxford, John Radcliffe Hospital, Level 6, West Wing, Oxford, United Kingdom; 2 Target Discovery Institute, Centre for Medicines Discovery, Nuffield Department of Medicine, University of Oxford, Oxford, United Kingdom; 3 Chinese Academy of Medical Science Oxford Institute, Nuffield Department of Medicine, University of Oxford, Oxford, United Kingdom

**Keywords:** Extracellular vesicle, Cerebrospinal fluid, Proteomics, Tetraspanin, Immunocapture

## Abstract

**Background:**

Due to its proximity to cells of the central nervous system, cerebrospinal fluid (CSF) is an important source of novel biomarkers for neurological diseases. Membrane-bound extracellular vesicles (EVs) are enriched for proteins of intracellular and membrane origin, implicated in the pathogenesis of some neurological diseases, and secreted into CSF. Proteomic profiling of CSF-EVs, however, is limited by the large volumes required for typical EV isolation protocols.

**Methods:**

We appraised the performance of tetraspanin (CD81, CD63, CD9)-based immunocapture for EV isolation from 200 to 1000 µL CSF sample and compared to size-exclusion chromatography (SEC). EVs were profiled by library-free data independent-acquisition (DIA) mass spectrometry to assess protein depth and abundance of specific EV markers and known co-isolates. Abundance and precursor peptide locations for potential neuronal-specific immunocapture targets described in the literature were also assessed.

**Results:**

Immunocapture was effective using CSF volumes as low as 200 µL, consistently detecting core EV markers and reducing relative levels of non-vesicular proteins such as Apolipoprotein B (APOB) and galectin 3 binding protein (LGALS3BP) compared with size-exclusion chromatography (SEC). Proteomic depth reached 811 ± 14 protein groups in EVs from 200 µL CSF, increasing to 1285 ± 224 when using feature alignment across runs with up to 1000 µL starting volume. These included eleven candidate biomarkers of neurological diseases that were detected in all preparation methods, with additional candidates detected by immunocapture only. Increased depth was observed for both transmembrane and secreted proteins using immunocapture compared with SEC, with proportional enrichment of transmembrane proteins.

**Conclusions:**

This work demonstrates the effectiveness of tetraspanin immunocapture for proteomic profiling of EVs in small volumes of CSF that can be adapted to use with cell-type-specific markers of choice.

**Supplementary Information:**

The online version contains supplementary material available at 10.1186/s12014-025-09579-9.

## Introduction

Cells, including those in the central nervous system (CNS), secrete extracellular particles into their external environment. The most heavily studied extracellular particles are extracellular vesicles (EVs), defined by the presence of a lipid-bilayer, which encompass vesicular particles produced by a variety of different mechanisms that may vary by cellular origin [1, 2]. A number of other nano-sized non-vesicular extracellular particles (NVEPs), including ribonucleoproteins (RNPs) such as argonaute complexes and vault particles, exomeres, supermeres, and single protein aggregates are also recognised [3–7]. Particularly in biofluids such as blood as cerebrospinal fluid (CSF), highly abundant particles such as high-, intermediate-, low- and very-low-density lipoprotein (HDL, IDL, LDL and VLDL) are also present and outnumber EVs by several orders of magnitude [5, 8].

EVs have attracted great attention as a novel source of fluid biomarkers in many neurological diseases, particularly neurodegenerative diseases such as Alzheimer’s disease, Parkinson’s disease and amyotrophic lateral sclerosis [9]. Compared with whole CSF, which comprises primarily abundant secreted proteins, CSF EVs have distinct a protein composition that is enriched for proteins of intracellular and membrane origin [10]. This feature makes EVs attractive as a potential biomarker source, as they are more likely to illuminate intracellular alterations central to the disease process rather than downstream effects of immune activation or blood-brain barrier alterations [11–13].

Shotgun proteomics has proven to be a useful tool for biomarker discovery in neurodegenerative diseases, and accessing the EV proteome can enable increased profiling depth but relies on effective separation of EVs from lipoproteins and serum albumin [14–16]. Previous studies in CSF have adopted many different approaches including ultracentrifugation, precipitation and size exclusion chromatography (SEC), using 500 µL to 16 mL volumes, attempting to balance the trade-off between purity and volume requirements [10, 17–19]. Affinity-based methods, exploiting the lipid or protein surface composition of EVs to separate them from NVEPs and concentrate from bulk fluid in a single step, can potentially reduce the starting volume required. Tetraspanin proteins such as CD81, CD9 and CD63 are highly abundant on the membrane of EVs from a variety of sources and are enriched relative to NVEPs [3, 4, 20, 21]. This study aimed to test the use of CSF EV immunocapture using antibodies against tetraspanins in small volumes of CSF to determine its suitability for use in proteomic biomarker discovery.

## Materials and methods

### Experimental design

This study assesses the suitability of tetraspanin-based immunocapture (CD81, CD63, CD9 antibody mix) as a methodology for enrichment of EVs from CSF for proteomic biomarker discovery, comparing against SEC as an existing standard. Initial characterisation experiments shown for SEC (Nanoparticle tracking analysis, immunoblotting, transmission electron microscopy) are for a 5 mL volume in a single experiment due to the large volumes required for replicates. Proteomics was performed on technical replicates of immunocapture from a pool of CSF (made up of ten individuals), with four replicates for each of three different starting volumes (200 µL, 500 µL, 1000 µL). These were compared against SEC, for which a single isolation from 2 mL CSF was carried out from the same pool and split into four replicates for protein digestion and subsequent proteomics, such that each SEC replicate is representative of 500 µL of starting CSF. Data analysis was carried out using match between runs (MBR) across either each sample preparation group (*n* = 4 for each) or across the entire dataset (*n* = 16). Outlying samples were excluded based on number of protein groups identified, either high count combined with evidence of cellular contamination (GOLGA2/GM130), or low protein count and in which one of the immunocapture target proteins was not detected. Assessment of proposed EV biomarker candidates for neurological diseases used all proteins listed in the International Society for Extracellular Vesicles CSF Task Force summary publication [22].

## CSF processing

CSF samples were obtained from individuals undergoing lumbar puncture at Oxford University Hospitals NHS Foundation Trust for routine care purposes. All participants provided informed consent. Diagnoses included idiopathic intracranial hypertension, post-infectious thoracic myelitis, relapsing-remitting multiple sclerosis and meningioma, including both male and female participants ranging from 27 to 57 years of age. Samples used in this study received ethical approval from Ethics Committee South Central–Oxford C (references 09/H0606/5 + 5 and 19/SC/0173). Lumbar punctures were performed in either the morning or afternoon, without fasting, using 22G NRFit Sprotte needle and 1% Lidocaine anaesthetic to collect up to 25 mL CSF per patient into sterile polypropylene tubes (Sarstedt). All samples were confirmed to be clear by visual inspection, before centrifuging within two hours of sampling at 2300 *g* for 10 min at 4 °C. Samples were then aliquoted and frozen at −80 °C in polypropylene storage tubes. For proteomics, aliquots of CSF from ten different individuals were thawed on wet ice and combined to produce a single pooled sample from which all technical replicates were derived (see Supplementary Table 1 for subject details). CSF underwent two freeze-thaw cycles before EV isolation.

## Size exclusion chromatography of extracellular vesicles

2–5 mL of pooled CSF was concentrated to 100 µL using an Amicon Ultra-15 10 kDa centrifugal filter (Merck Millipore) at 3500 *g*. For immunoblotting and TEM a starting CSF volume of 5mL was used, and for proteomic analysis 2mL starting CSF volume (corresponding to 500 µL per replicate of proteomics). The sample was injected onto a Tricorn 5/150 column with 3 mL bed volume packed with sepharose 4 fastflow (mean particle size 90 μm, exclusion limit 3 × 10^7^ M_r_) and eluted at 0.3 mL/minute in phosphate buffered saline pH 7.4 without calcium and magnesium (PBS) using an ÄKTA Go fast protein liquid chromatography system (Cytiva). 200 µL fractions were collected. For proteomics, two particle-containing fractions were pooled and then split into four 100 µL samples for independent lysis replicates. 100 µL 10% sodium dodecyl sulfate in 100 mM triethylammonium bicarbonate (TEAB) was added to each and incubated on an orbital shaker at 1000 rpm at room temperature for 20 min.

## Nanoparticle tracking analysis

Particle counting was carried out on intact EVs using the Zetaview Nanoparticle Tracking instrument (Particle Metrix), calibrated with silica 100 nm microspheres (Polysciences Inc., Philadelphia, United States). Samples were diluted to 100–200 particles per frame in PBS. Data was acquired at room temperature with settings: sensitivity 80, shutter 100, frame rate 30, cycles 2, minimum brightness 20, maximum size 1000, minimum size 10, minimum tracelength 15, at all 11 positions and analysed using zetaview software (version 8.05.12 SP2).

## Transmission electron microscopy

EVs were incubated for 2 min on glow discharged (20 s at 15 mA) carbon 300 mesh copper grids (Agar Scientific, AGS160-3) pre-treated with 0.1% poly-L-lysine solution for 5 min. Grids were negatively stained with 2% uranyl acetate for 10 s before blotting dry. Imaging was performed using an FEI Tecnai 12 transmission electron microscope at 120 kV using a Gatan OneView CMOS camera.

### Immunoblotting

Immunoblotting was carried out using a tris-glycine gel system for separation (Novex, Invitrogen). EVs were lysed directly in 2x SDS sample buffer with (for syntenin-1) or without (for tetraspanins) NuPAGE dithiothreitol reducing agent (Invitrogen), and frozen at −80 °C. Before loading, samples were heated to 65 °C for 12 min. Samples were separated on WedgeWell 8–16% tris-glycine mini gels in tris-glycine SDS running buffer alongside Spectra multicolour broad range protein ladder (Fisher Scientific) for 90 min at 150 V. Proteins were dry transferred to 0.2 μm PVDF membranes using an iBlot2 system (20 V for 1 min, 23 V for 4 min, 25 V for 1 min). After blocking in 5% milk in 0.1%-TBS-Tween for 1 h, membranes were incubated overnight with primary antibodies; CD81 (Thermofisher 10828D), CD9 (Millipore, CBL162), CD63 (Thermofisher 10628D) or Syntenin-1 (Abcam, 133267) at 1:1000 in 1% milk/0.1% TBS-Tween. After washing three times in 0.1%-TBS-Tween, membranes were incubated with rabbit or mouse horseradish peroxidase-labelled secondary antibodies (VWR International) at 1:10,000 for 1 h at room temperature. Detection was carried out with ECL Prime (Cytiva) on ChemiDoc MP (BioRad).

## Immunocapture of extracellular vesicles

EV immunocapture was performed using 2.8 μm magnetic M-270 Epoxy Dynabeads (Invitrogen 14311D) covalently conjugated to a mix of CD81 (Thermofisher 10828D), CD9 (Millipore, CBL162) and CD63 (Thermofisher 10628D) antibodies in 2:1:1 ratio or hemagglutinin (HA; Invitrogen 26183) antibody as negative control. 4 µg total antibody was added per 1 mg of dynabeads and incubated overnight at 37 °C on an orbital shaker at 800 rpm. Beads were washed as per manufacturer’s protocol, before addition of SynBlock ELISA Blocking Buffer (Enzo, ICT-641) and further washing with PBS. Each independent capture replicate used 200–1000 µL pooled CSF sample made up to a total volume of 1000 µL in PBS, with 0.5 mg conjugated beads, in 2 mL low protein binding Eppendorf tubes. Samples were rotated overnight at 4 °C. Before elution, beads were washed once with 0.05% PBS-Tween and once with PBS. Samples were eluted in 50 µL 5% sodium dodecyl sulfate in 100 mM TEAB on an orbital shaker at 1000 rpm at room temperature for 20 min. Four independent immunocaptures were carried out per CSF volume.

## Preparation of samples for proteomics

EV samples were digested for proteomics using a suspension trapping method (Protifi, Fairport, NY). After lysis, protein was reduced and alkylated (5 mM dithiothreitol and 20 mM iodoacetamide respectively) for 30 min at room temperature each in the dark. Phosphoric acid was used to acidify the samples to a final concentration of 2.5%, which were then mixed with six volumes of binding buffer (90% aqueous methanol with 100 mM TEAB). Samples were applied to S-trap micro columns and centrifuged at 4,000 *g*, repeating for the full sample volume. Bound protein was washed five times with 150 µL binding buffer. 400 ng trypsin/Lys-C mix was added to each column in 40 µL 50 mM TEAB. Columns were incubated for one hour at 37 °C in a water-saturated atmosphere before addition of 40 µL more 50 mM TEAB and overnight incubation. To elute peptides, 40 µL 50 mM TEAB was added before centrifugation at 4000 *g* for one minute, followed by successive addition of 40 µL 0.2% aqueous formic acid and 40 µL 50% aqueous acetonitrile with 0.2% formic acid, centrifuging at 1500 *g* after each addition. Peptides were frozen, then thawed for drying and resuspension in 60 µL 2% acetonitrile/0.1% formic acid before analysis.

### Mass spectrometry

Liquid chromatography–tandem mass spectrometry (LC–MS/MS) was used to analyse the tryptic peptides using the Vanquish Neo UHPLC connected to the Orbitrap Ascend Mass Spectrometer equipped with a high-field asymmetric ion mobility spectrometry (FAIMS) Pro Duo interface (all Thermo Fisher Scientific) with an EASY-Spray Source. The Vanquish Neo was operated in “Trap and Elute” mode using a PepMap Neo trap (5 μm, 300 μm x 5 mm; Thermo Fisher) and separated using an EASY-SPRAY PepMapNeo column (50 cm x 75 μm, 1500 bar; Thermo Fisher) heated at 50 °C. Mobile phase A and B were 0.1% Formic acid in water (LC-MS Optima grade) and 0.1% Formic acid in Acetonitrile (LC-MS Optima grade), respectively. 16% of tryptic peptides from each sample were injected, trapped and separated over a 60 min gradient, going from 2 to 18% B in 40 min, to 35% in 20 min, up to 99% B in 1 min and then staying at 99% for 14 min. The flow rate was maintained at 300 nL/min throughout the gradient. The FAIMS Pro Duo was operated in standard resolution mode with carrier gas flow rate of 3.8 L/min and compensation voltage set to −45. Data acquisition was carried out in data-independent mode (DIA) similar to previous studies [23–25]. MS1 scans were collected in the orbitrap at a resolving power of 45 K at m/z 200 over m/z range of 350 to 1,650 m/z. The MS1 normalized automatic gain control was set at 125% (5e5ions) with a maximum injection time of 91 ms and a radio frequency lens at 30%. DIA MS2 scans were then acquired using the tMSn scan function at 30 K orbitrap resolution over 40 scan windows with variable width, with a normalized automatic gain control target of 1,000%, maximum injection time set to auto and a 30% collision energy.

Library-free analysis of raw data files was carried out using DIA-NN (v1.8.1) against the UniprotSwissprot reviewed human proteome (downloaded July 2023 and containing 20,426 sequences). Default settings were used to generate label free quantitation data [26]. Settings used were 1% peptide false discovery rate, trypsin as proteolytic enzyme with one missed cleavage allowed, inclusion of N-terminal excision, Cysteine carbamidomethylation enabled as a fixed modification and oxidation of methionine as variable modifications. The match between runs (MBR) algorithm within DIA-NN was used for feature alignment across MS runs to reduce missing peptide identifications by the transfer of MS/MS peptide identifications in one run to other runs with peptide signal of the same mass and retention time. Data were analysed both with MBR across all 16 samples, or for each group of technical replicates separately.

### Data processing

Data files were filtered to include only proteins which appeared in at least three of four replicates of at least one of the four different groups, and then log_2_ transformed. Data were either left non-normalised, or normalised to the mean abundance of CD81, CD63 and CD9 tetraspanin proteins. Aside from comparison of protein depth, all analysis was carried out on dataset deriving from analysis with MBR applied across all 16 samples. Coefficients of variation were calculated from non-logged abundance data after normalisation to mean abundance of CD81, CD63 and CD9 tetraspanin proteins. Three outlying samples were excluded based on number of proteins identified, two with high protein count with evidence of cellular contamination (GOLGA2/GM130) and one with low protein count and in which CD63 was also not detected (Supplementary Fig. 2 A).

### Gene ontology

Overrepresentation analysis was carried out using ClusterProfiler (v 4.6.2) in R (v 4.2.0), using either analysis of all gene ontology terms (Biological Process, Cellular Component and Molecular Function) or user-defined categories from the Human Protein Atlas (proteinatlas.org) [27, 28] as of 5th January 2025. For gene ontology, background was defined as all proteins detected in the dataset after the exclusion of three outlying samples. Dotplots show the ten most significant terms after Benjamini-Hochberg p-value adjustment and use of “simplify” method to remove redundant terms. For cell type marker enrichment, genes defined as cell-type enriched by the Human Protein Atlas (at least four-fold higher mRNA level that cell type compared to any other cell type) for glial, neuronal, epithelial, blood and immune cells were downloaded from the Human Protein Atlas. 1064 genes classed as regionally elevated within the brain according to the Human Protein Atlas were used for analysis of brain regions. As background a combination of all 3174 proteins detected in a high-depth proteomic study of whole CSF [29] and proteins in our CSF EV dataset (after exclusion of outliers), giving a total of 4429 proteins. Venn diagrams were produced using VennDiagram package (v 1.7.3). Upset plots were produced using UpsetR package (v 1.4.0).

### Statistical analysis

Hierarchical clustering was carried out in R (v 4.2.0) using pheatmap package (v 1.0.12), clustering by “complete” method using Euclidian distance for rows. Two-way ANOVA followed by TukeyHSD posthoc test, or Kruskal-Wallis test followed by pairwise Wilcoxon-rank sum posthoc test with Benjamini-Hochberg correction, were used to analyse differences between experimental groups (R v 4.2.0). Statistical significance cut-off was set at 0.05. Spearman correlation for proposed neuronal EV capture targets was carried out using pairwise complete observations, excluding GAP43 and ATP1A3 to ensure at least three complete observations for each protein pair. Correlation plots were produced using corrplot package (v 0.95). Pairwise differential abundance analysis was carried out in R using limma (v 3.54.2) on protein groups containing no missing values for the pairwise comparison. Empirical Bayes moderation was applied to obtain moderated t-statistics due to the small sample size (*n* = 3 per group), with Benjamini-Hochberg method for false-discovery rate (FDR) correction, and a cut-off of FDR-adjusted *p* < 0.05.

### Mapping of precursor peptides

Mapping of precursor peptides was carried out using custom code in R. Canonical protein sequence and associated topological data (TRANSMEM, TOPO_DOM) were imported from Uniprot (www.uniprot.org/) as a reference [30], as of 5th January 2025. For each protein of interest, precursor peptides annotated to this protein were filtered to exclude non-proteotypic peptides and aligned against the reference sequence.

### Protein type

Protein type was defined by extracting subcellular location data from Uniprot (www.uniprot.org/) for all detected proteins [30]. Proteins were defined as “Secreted” if matching to string “Secreted” and not followed by “extracellular exosome” or “extracellular matrix”. “Single-pass”, “Multi-pass”, “Lipid-anchor” and “Peripheral” were used as additional search strings for membrane proteins. All proteins not matching any of these terms were designated as “Other”. Proteins matching to multiple classification types were grouped as “multiple annotations.”

## Results

### Isolation of tetraspanin-positive extracellular vesicles in CSF using SEC and immunocapture

**Fig. 1:**
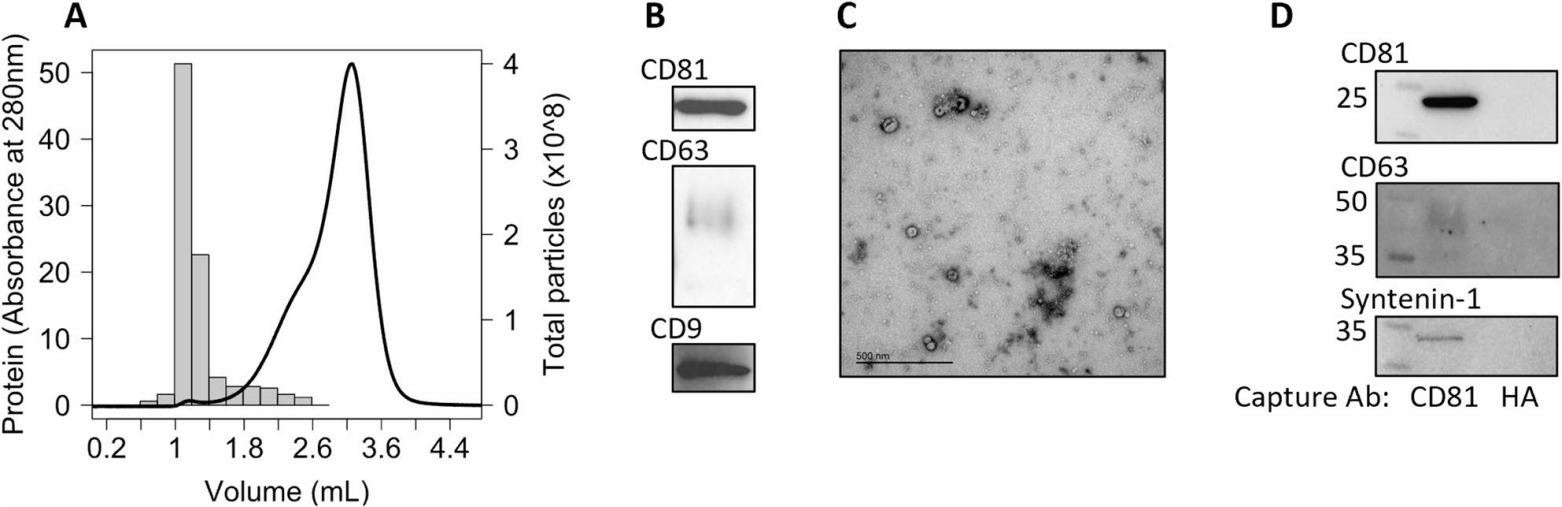
Characterisation of EVs enriched by size exclusion chromatography or immunocapture. (**A**) Isolation of EVs from cerebrospinal fluid by size-exclusion chromatography (SEC) using fast protein liquid chromatography; line and left y-axis shows UV absorbance at 280 nm with peak at 1–1.4.4 mL, indicative of protein concentration, bars and right y-axis show NTA particle counts from 200 µL fractions with peak at 1–1.4.4 mL (*n* = 1). (**B**) Immunoblotting of SEC-EVs from pooled elution volume 1–1.4.4 mL. (**C**) Representative TEM of negatively stained SEC–EVs from pooled elution volume 1.0–1.4.0.4 mL. (**D**) Immunoblotting for EVs enriched by immunocapture using CD81 antibody or hemagglutinin (HA) isotype-matched negative control. Ab, antibody.

Size-exclusion chromatography of a concentrated 5 mL CSF sample was undertaken with a 3 mL volume column containing sepharose 4 fast flow resin. 280 nm absorbance demonstrated a small peak at an elution volume of 1 −1.4 mL, corresponding to the terminal portion of the void volume, prior to bulk protein eluting from 1.6 mL and accompanied by a distinct peak in particle number by nanoparticle tracking analysis (NTA; Fig. 1A). Fractions from the particle peak were found to be positive for tetraspanins CD63, CD9 and CD81 by immunoblot, indicating the presence of EVs (Fig. 1B; Supplementary Fig. 1 A). Transmission electron microscopy indicated 60–80 nm cup-shaped particles with a morphology consistent with negatively stained membrane vesicles (Fig. 1C). Additional smaller structures around 40 nm were also observed, indicating a diverse population of nanoparticles within SEC samples.

Immunocapture against CD81 directly from whole CSF was able to precipitate material positive for the EV membrane marker CD63 and cytoplasmic marker syntenin-1 from 500 µL CSF, indicating capture of intact EVs (Fig. 1D). Further characterisation of immunocapture isolates using NTA and TEM was not possible due to the challenges of eluting EVs from beads without stringent detergent conditions that lead to membrane dissociation [31].

### Proteomic depth achieved by immunocapture versus size exclusion chromatography

**Fig. 2:**
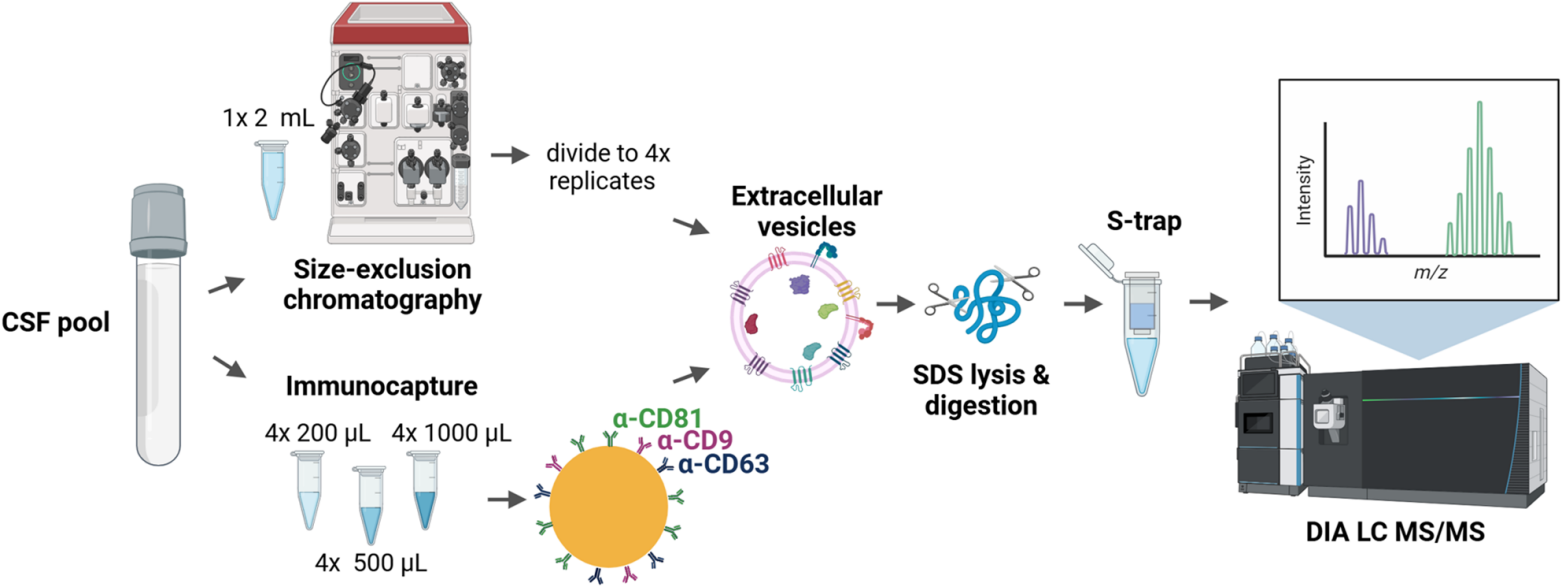
Experimental outline for proteomics of CSF extracellular vesicles. CSF, cerebrospinal fluid; SDS, sodium dodecyl sulfate; DIA LC MS/MS, data independent acquisition liquid chromatography tandem mass spectrometry. Created in https://BioRender.com

Since the study aimed to determine the suitability of CSF tetraspanin immunocapture for proteomic biomarker discovery, capture was performed using different CSF volumes, 200 µL, 500 µL and 1000 µL (four replicates each) from a single pool of CSF, using beads conjugated to a cocktail of three tetraspanin antibodies (CD9, CD63, CD81) to capture a broader range of EVs than CD81 alone (Fig. 2). Given the high relative abundance of CD81 in CSF EVs, a 2:1:1 ratio of CD81:CD63:CD9 was used **(**Supplementary Fig. 1 C**)** [21]. For comparison, a single isolation by SEC from 2 mL CSF was carried out and split into four replicates for protein digestion and subsequent proteomics.

**Fig. 3:**
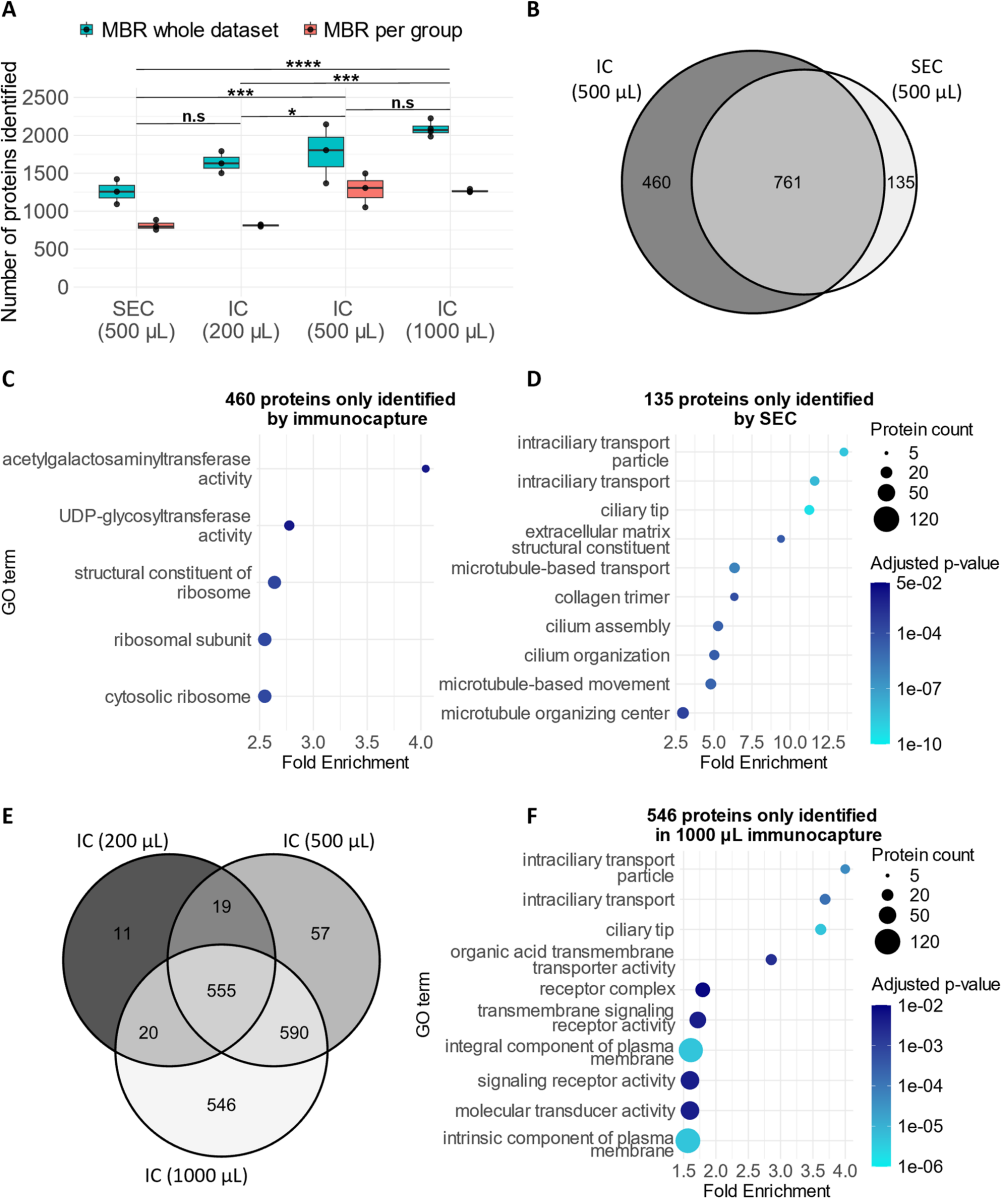
Comparison of proteins detected in SEC and immunocapture. (**A**) Total number of proteins identified in mass spectrometry with MBR applied across each group of four replicates, or on the whole dataset. Two-way ANOVA followed by Tukey’s posthoc test. n.s., non-significant; * *p* < 0.05; *** *p* < 0.001; **** *p* < 0.0001. **B**) Venn diagram for proteins identified in SEC and immunocapture from equivalent (500 µL) volumes, where protein groups are defined as present if detected in at least 3 of four replicates from analysis with MBR across the whole dataset. (**C**) and (**D**) Overrepresentation analysis of proteins identified solely in immunocapture or SEC respectively, showing up to the top ten most significant terms. (**E**) Venn diagram showing overlap between proteins identified by immunocapture in three different starting CSF volumes. (**F**) Overrepresentation analysis of proteins identified only from starting volume 1000 µL, showing the top ten most significant terms. IC, immunocapture; SEC, size-exclusion chromatography; GO, gene ontology; MBR, match between runs.

Depth of proteomic analysis was profiled in EV extracts initially by analysing each volume group separately in DIA-NN, applying match between runs (MBR) to each four replicates of a given CSF starting volume separately. After exclusion of three outlying samples, we detected 811 ± 14 protein groups from 200 µL starting CSF by immunocapture, significantly increasing to 1285 ± 224 from 500 µL to 1266 ± 18 from 1000 µL (Fig. 3A; Supplementary Fig. 2 A); SEC of a 500 µL- equivalent CSF starting volume yielded 812 ± 66 protein groups.

Applying MBR across all samples increased the number of protein groups detected in all CSF starting volumes: 1641 ± 145 protein groups for 200 µL, 1772 ± 390 for 500 µL, 2087 ± 101 for 1000 µL, and 1266 ± 17.8 for SEC (Fig. 3A), indicating that the gains in depth obtained through increasing CSF starting volume for immunocapture might be somewhat negated by increasing the number of samples analysed.

To compare the functional enrichment of proteins detected by immunocapture versus SEC, proteins were defined as present in a given CSF starting volume or by SEC if detected in at least three of the four replicates (when MBR was applied across the whole dataset). Comparing SEC and immunocapture from a 500 µL equivalent starting volume, there were 761 protein groups in common between the two methods, with 460 detected only in immunocapture and 135 only in SEC (Fig. 3B). GO overrepresentation analysis of the non-overlapping proteins showed that immunocapture samples was enriched for ribosomal terms (Fig. 3C), whilst SEC samples were enriched for microtube, extracellular matrix, and ciliary related proteins (Fig. 3D). Overlapping terms were most enriched for terms relating to coagulation and immunity (Supplementary Fig. 2B).

Proteins detected by immunocapture across the three different starting CSF volumes were then compared. Almost all proteins reproducibly detected from 200 µL starting CSF were also detected from larger starting volumes (Fig. 3E). The 546 additional proteins that were detected only from the largest 1000 µL starting volume were most significantly enriched for “integral component of plasma membrane”, alongside additional terms relating to ciliary proteins (Fig. 3F), suggesting that the additional proteins detected are likely to be of predominantly vesicular origin, rather than an increase in the number of co-isolated components.

### Enrichment of extracellular particle markers

**Fig. 4:**
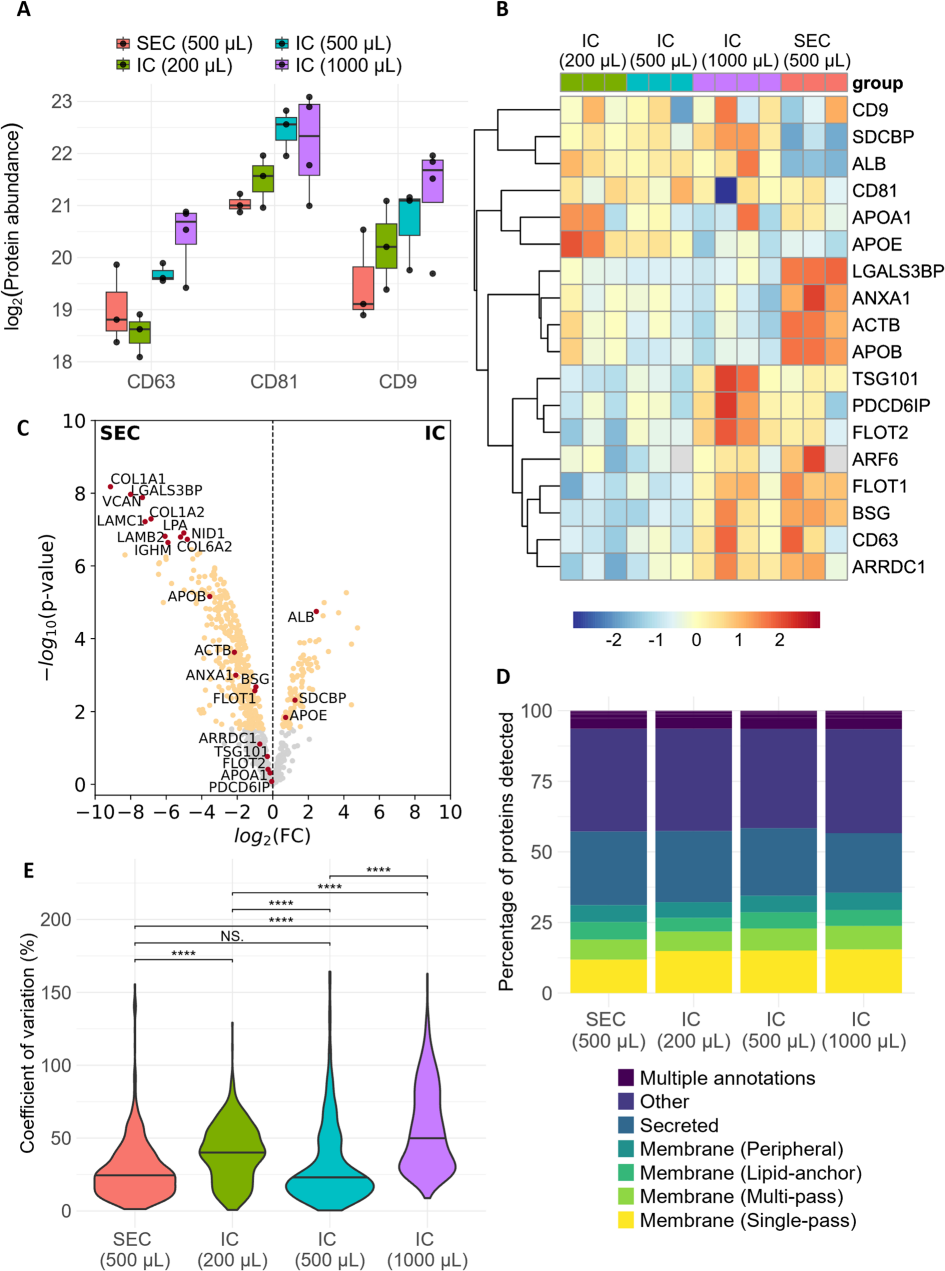
Proteomics comparison of extracellular particle marker proteins. (**A**) Abundance of three immunocapture target tetraspanins in mass spectrometry data. (**B**) Heatmap for proteins commonly associated with endosomal-derived EVs (SDCBP, PDCD6IP, TSG101, FLOT1, FLOT2), plasma-membrane shed EVs (ANXA1, ACTB, ARF6, BSG, ARRDC1), co-isolated components (APOA, APOE, APOB, LGALS3BP, ALB) and tetraspanins (CD81, CD9, CD63) after normalisation to average abundance of tetraspanin immunocapture targets. (**C**) Volcano plot showing differential abundance of all proteins detected consistently in 500 µL starting volume samples after outlier removal (n=3 per condition, total proteins 777). (**D**) Mean percentage of proteins detected in each sample within each protein type according to Uniprot. Full data shown in Supplementary Table 3. IC, immunocapture; SEC, size-exclusion chromatography. (**D**) Coefficients of variation for 699 proteins which had no missing values across the dataset. Kruskal–Wallis test followed by pairwise Wilcoxon rank-sum posthoc test with Benjamini-Hochberg correction. ****, p < 0.0001; NS, non-significant.

The relative abundance of specific markers associated with different populations of extracellular particles were then analysed to assess the yield and specificity of EV isolation. Firstly, the abundance of the tetraspanins CD81, CD9, CD63 (against which the immunocapture antibodies were targeted) were compared in non-normalised data, demonstrating that abundance for all three proteins was CSF volume-dependent (Fig. 4A). Importantly, in this non-normalised data all three volumes showed higher abundance of CD81 and CD9 compared to SEC, demonstrating the effectiveness of the immunocapture technique for increasing yield.

Abundance of marker proteins thought to be indicative of endosomal-derived EVs was then analysed relative to average abundance of target tetraspanins. Syndecan binding protein (syntenin-1; SDCBP), programmed cell death 6 interacting protein (ALIX, PDCD6IP), tumour susceptibility 101 (TSG101), flotillin 1 (FLOT1) and flotillin 2 (FLOT2) were consistently detected in all samples, (Fig. 4B; Supplementary Fig. 3 A). Differential abundance analysis was carried out on all proteins consistently detected in immunocapture versus SEC from 500 µL samples to assess relative purity, demonstrating an enrichment of syntenin-1 (log2FC = 1.25; FDR = 0.0048; Fig. 4C). Other proteins within the tetraspanin family, including CD82, TSPAN4 and TSPAN14, were also detected and, with the exception of CD151, were of highest abundance in immunocapture samples in normalised data (Supplementary Fig. 3B). Some protein markers more typically associated with plasma membrane-shed EVs, annexin A1 (ANXA1), basigin (BSG) and actin (ACTB), were also consistently detected, but at reduced abundance in immunocapture relative to SEC (ANXA1 log2FC = −2.08, FDR = 0.0031; BSG log2FC = −0.95, FDR = 0.0021; ACTB log2FC =−2.16, FDR = 0.0011;Fig. 4B and C; Supplementary Fig. 3 C).

Similarly to blood, whole CSF contains a range of very highly abundant proteins, including albumin (ALB), Apolipoprotein B (APOB, the major protein constituent of LDL, IDL and VLDL particles), Apolipoprotein A1 (APOA1, a major protein constituent of HDL), Apolipoprotein E (APOE, the major cholesterol transporter in the brain), and LGALS3BP (a secreted glycoprotein reportedly highly abundant in exomeres) [4, 6, 25, 29]. These proteins are common co-isolates of EVs so their abundance in SEC versus immunocapture was also compared to assess purity. APOB and LGALS3BP were both highly depleted in immunocapture relative to SEC (LGALS3BP log2FC = −8.00, FDR < 0.0001; APOB log2FC = −3.55, FDR < 0.0001, indicating that they are not integral components of tetraspanin-positive EVs (Fig. 4B and C; Supplementary Fig. 3D). Collagens (COL1A1, COL1A2, COL6A2) and other extracellular matrix components (VCAN, LAMC1, LAMB2) were also amongst the most highly depleted proteins in immunocapture **(**Fig. 4C). APOA1 was very highly abundant and showed no significant difference in abundance between immunocapture and SEC, whilst APOE was slightly enriched by immunocapture (log2FC = 0.72, FDR = 0.027; Fig. 4C, Supplementary Fig. 3D**)**. Albumin, as a key marker of soluble protein and the most abundant CSF protein, was increased in immunocapture samples (log2FC 2.45, FDR < 0.0001; Fig. 4C).

The relatively higher abundance of albumin seen with immunocapture might indicate that the additional proteins detected by immunocapture reflect co-isolated soluble protein rather than being of vesicular origin. Protein localisation was therefore assessed according to Uniprot annotation of proteins as membrane or secreted. By immunocapture, there was a volume-dependent increase in the number of proteins detected in all classes; peripheral membrane, single-pass membrane, multi-pass membrane and secreted, alongside “other” (cytoplasmic or no annotation; Supplementary Table 2). As a proportion of total proteins detected, secreted proteins were depleted and membrane-spanning proteins enriched in immunocapture samples relative to SEC (Fig. 4D; Supplementary Table 3), suggesting that immunocapture increases depth of the vesicular proteome as opposed to co-isolated soluble protein.

In order to assess the variability in abundance of the proteins quantified by each method, the coefficient of variation (CV) for all proteins found consistently in all samples (699 protein groups) were compared. Median CV was lowest for SEC (median 24.2%, IQR 14.2–37.1), which was expected given that these were replicates at the level of digestion, rather than at purification (Fig. 4E). For immunocapture, overall median CV for 500 µL (median 22.0%, IQR 13.6–43.6) was comparable to SEC (*p* = 0.89), with higher median CV in 200 µL (median 39.8%, IQR 24.5–53.7, *p* < 0.0001), and highest for 1000 µL (median 49.6%, IQR 31.2–77.3, *p* < 0.0001; Fig. 4E). These data indicate that the variability is not directly related to CSF starting volume.

**Fig. 5:**
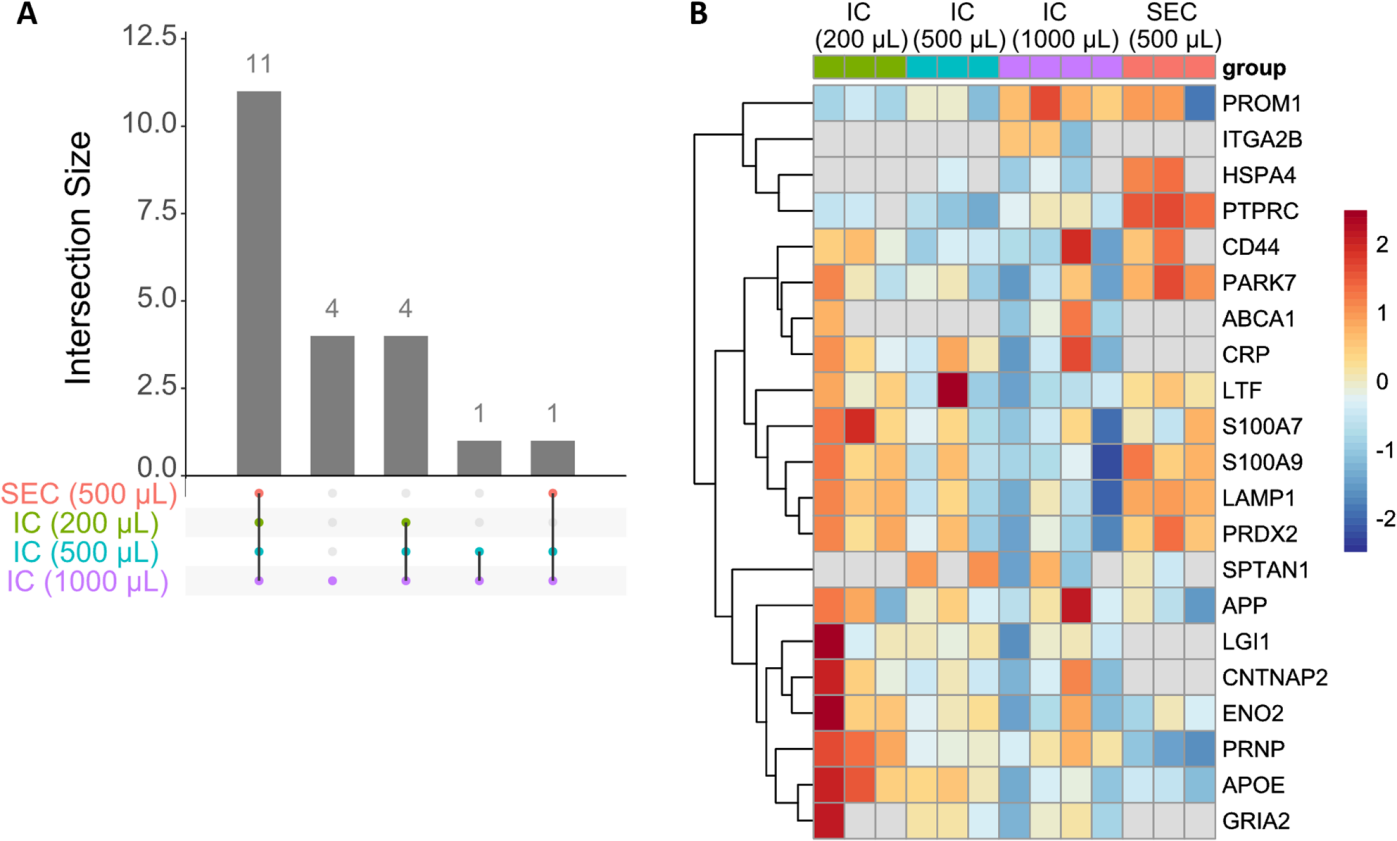
Candidate biomarkers for neurological diseases that are detected in CSF extracellular vesicles. (**A**) Upset plot showing proposed neurological disease biomarker candidates listed in Sandau et al. (2024) that are reproducibly detected in CSF EVs. Proteins shown as detected if present in at least three replicates of the group. 36 other proteins did not reach this filtering threshold. (**B**) Heatmap showing abundance of neurological disease biomarker candidates after normalisation to average abundance of tetraspanin immunocapture targets. Grey colour indicates samples where protein is not detected.

To further assess the suitability of the immunocapture method for biomarker discovery, a panel of 57 previously described EV-localised biomarker candidates of neurological diseases were assessed in the dataset [22]. Of these 57 proteins, 21 were reproducibly detected in at least one of the four sample groups; 11 of these in SEC and immunocapture from all CSF volumes (Fig. 5A). Immunocapture enabled the detection of four additional candidate biomarkers in all CSF volumes, with a further six at higher CSF start volumes. No proteins were detected only in SEC. Proteins detected only by the immunocapture method included GRIA2, CNTNAP2 and LGI1, all proteins which exhibit relatively high specificity to cells of CNS-origin (Fig. 5B). These data indicate that the tetraspanin-based immunocapture method is suitable for the detection and quantification of currently known biomarker candidates.

**Fig. 6:**
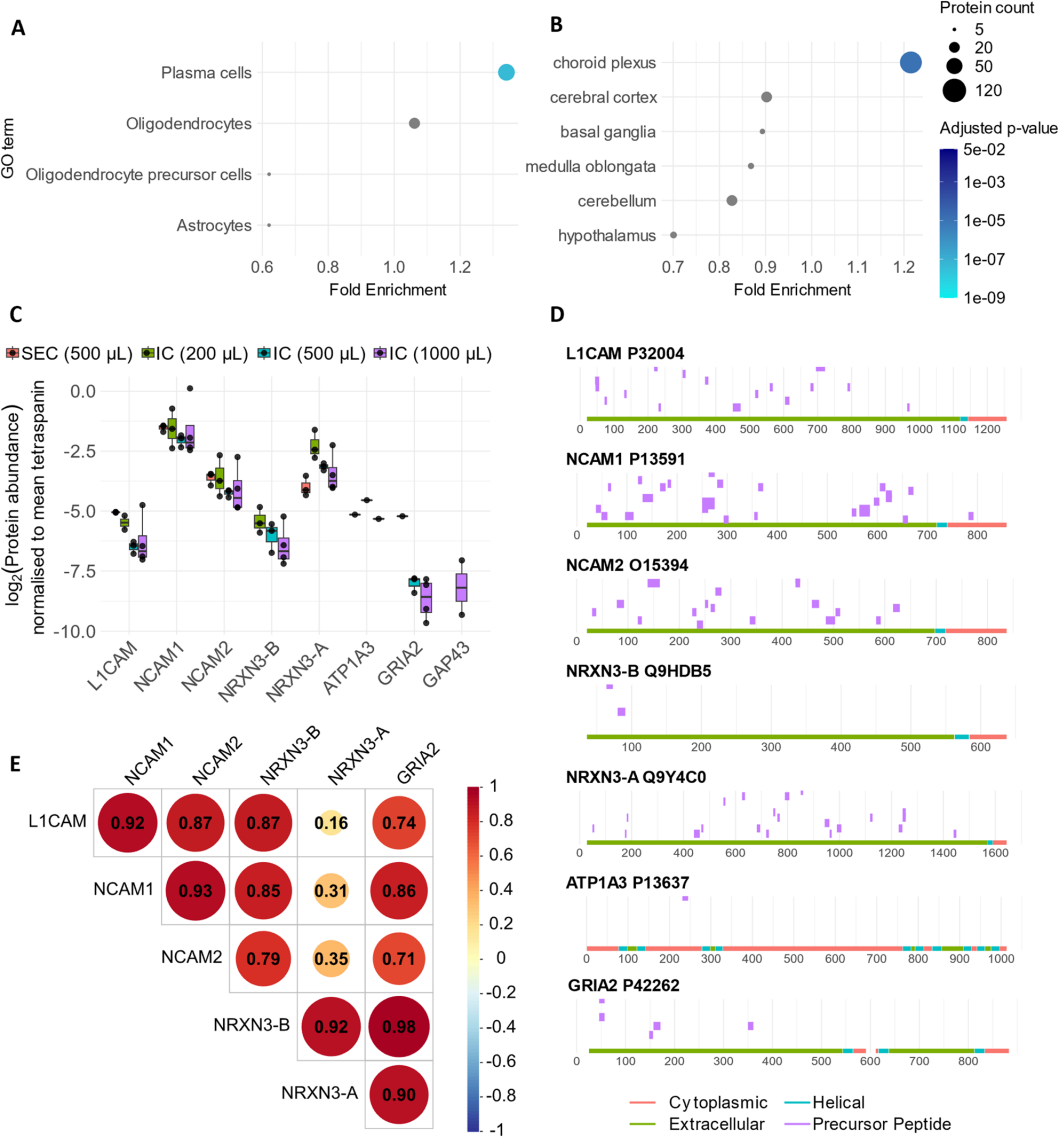
Assessment of markers of brain and neuronal origin in CSF EVs. (**A**) Overrepresentation analysis of markers of different cellular origins for proteins detected anywhere in the CSF EV dataset against a background of all known CSF proteins. (**B**) Overrepresentation analysis of markers of specific brain region for proteins detected anywhere in the CSF EV dataset against a background of all known CSF proteins. Grey points indicate non-significance *p* > 0.05. (**C**) Total protein abundance of literature-identified neuronal EV immunocapture targets in tetraspanin-normalised mass spectrometry data. (**D**) Mapping of precursor peptides for literature-identified neuronal EV immunocapture targets from mass spectrometry against canonical full-length sequence and topological data (cytoplasmic, extracellular and helical transmembrane domains) from Uniprot. No topological data available for GAP43. (**E**) Spearman correlation matrix for neuronal EV immunocapture targets. GO, gene ontology.

Given the ability to detect biomarker candidates of interest, cell type enrichment analysis was then performed to ascertain the origin of the EVs isolated. In the whole CSF EV dataset compared to a background of all proteins known in CSF [29] there was significant enrichment of plasma cell proteins only (fold enrichment 1.34, *p* < 0.0001; Fig. 6A). All proteins within this set corresponded to immunoglobulins. Overrepresentation of specific brain regions was also sought, with significant enrichment for choroid plexus only (the secretory tissue primarily responsible for CSF production; fold enrichment 1.22, *p* < 0.0001; Fig. 6B).

Multiple different marker proteins have been proposed to enable enrichment of rare EV populations, including potential neuronal-specific transmembrane proteins such as L1 cell adhesion molecule (L1CAM), neural cell adhesion molecule 1 (NCAM1), neural cell adhesion molecule 2 (NCAM2), neurexin 3 (NRXN3), ATPase Na+/K + transporting subunit alpha 3 (ATP1A3), growth associated protein 43 (GAP43), neuroligin 3 (NLGN3), microtubule associated protein 1B (MAP1B) and glutamate ionotropic receptor AMPA type subunit 2 (GRIA2) [32–38]. However, there remain questions about both the neuronal specificity of these proteins, and the existence of soluble protein forms resulting from proteolytic cleavage by proteases [38–40]. L1CAM, NCAM1 and NCAM2 were detected in the majority of samples and were most abundant in SEC EVs in tetraspanin-normalised data (Fig. 6C). GRIA2 and GAP43 were detected in immunocapture samples only (eight and two samples respectively; Fig. 6C). Two different isoforms of NRXN3 were detected, the higher abundance α-isoform (containing epidermal growth factor-like sequences) and β (lacking epidermal growth factor-like sequences), but with precursor peptides specific to the β form detected only in the immunocapture samples **(**Fig. 6C).

To provide further evidence for the presence of membrane-bound and soluble forms of putative neuronal marker proteins within the EV proteomic dataset, precursor peptides were mapped to canonical reference sequences and annotated topological domains. For L1CAM, NCAM2, NRXN3 and GRIA2, only peptides mapping to extracellular regions were detected, whilst for NCAM1 and ATP1A3, one precursor peptide each mapped to a cytoplasmic region of the protein (Fig. 6D). No topological information was available for GAP43. For L1CAM, NCAM2 and both isoforms of NRXN3, peptide coverage in CSF EV data was similar to that of whole CSF prepared for proteomics using the same SDS-based method (Supplementary Fig. 4) [25]. However, ATP1A3, GRIA2 and the cytoplasmic peptide for NCAM1 were not detected in whole CSF data, indicating that they might be more abundant in the CSF EVs [25]. Finally, the abundance of these neuronal proteins was correlated with each other, which demonstrated a strong inter-relationship between most of these putative neuronal marker proteins with correlation coefficients ranging from 0.16 to 0.98, indicating a likely similar source (Fig. 6E).

## Discussion

The development of new sample preparation, mass spectrometry instrumentation and data processing methodologies has enabled continued increases in the proteomic depth and reproducibility that is possible using small volumes of biofluids for biomarker discovery [16, 26, 29, 41–45]. While mass spectrometry of CSF EVs can access a different portion of the CSF proteome, a key limiting factor for clinical proteomics is the far larger sample volume required [14, 17]. This study examined the performance of tetraspanin immunocapture of CSF EVs compared with SEC purified CSF EVs. With dataset-wide MBR we were able to achieve a total depth of 2087 ± 101 proteins from EVs immunocaptured from 1000 µL starting CSF using a 60-minute gradient, and 1641 ± 145 from only 200 µL, showing very high protein depth relative to starting volume compared to other recent studies, with reproducibility of quantification that is at least comparable to SEC [17, 37, 46]. Furthermore, it confirms detection of a range of known candidate biomarkers of neurological diseases in these low volumes [22].

Using immunocapture, a higher proportion of proteins identified contained transmembrane domains, giving higher confidence that they are of truly vesicular origin. However, immunocapture also enabled the detection of higher numbers of secreted proteins when compared with SEC. This may be a reflection of less effective exclusion of abundant soluble proteins (or their non-specific association with beads), differences in the exclusion of particulate contaminants (such as lipoprotein particles) or, since the charged surface of EVs may attract a “corona” of soluble proteins, that the increased yield in immunocapture attracts and concentrates low abundance soluble proteins to better enable their detection [9, 47, 48].

Key to increasing the depth in biofluid mass spectrometry is the reduction of very highly abundant proteins which mask the detection of low abundance proteins. In a meta-analysis of plasma EV studies, the proteome of “EVs” isolated by polymer-based precipitation methods comprised up to 70% apolipoprotein and serum albumin, contrasting with density gradient ultracentrifugation in which this fraction was under 10% [15]. Our data indicate that in CSF, tetraspanin immunocapture effectively depletes APOB (expected to be contained within LDL and VLDL particles) and LGALS3BP, but not APOA1 (a marker of HDLs) or APOE. Spike-in experiments in plasma have demonstrated that APOA1 very readily adsorbs to the vesicular surface, possibly due to specific interactions with binding partners such as ABCA1, or charge-based interactions that are stronger than those of APOB [47, 49–51].

Recent research has suggested that some proteins or RNAs that have been attributed as EV cargoes, such as miRNAs and glycolytic proteins, may instead derive primarily from NVEPs and lipoproteins [3, 4, 52–54]. LGALS3BP is a very highly abundant protein in CSF and in non-vesicular “exomeres” [4, 6, 11, 55]. By TEM we visualised 40 nm particles in SEC samples that were reminiscent of ring-like aggregates of LGALS3BP that have been reported in the literature [56, 57]. The strong reduction of LGALS3BP in our data indicates that immunocapture may offer an approach by which to better separate different nanoparticle populations, and to define the extracellular secretion pathway of molecules of interest, which may have importance to understanding of both disease pathogenesis and reproducible quantification in biofluids.

Despite tetraspanin immunocapture providing higher overall protein coverage than SEC in CSF EVs, the data show that there is a likely a difference in EV subpopulations, with gene ontology indicating better coverage of ciliary and extracellular matrix components in SEC compared to tetraspanin immunocapture; in addition to higher relative abundance of proteins such as ANXA1 which are typically associated with plasma-membrane-shed EVs [5]. Further work will be required to assess whether these differences associated with enrichment methodology in CSF are reproduced in other biofluids such as blood and urine.

Accurate quantification in proteins in CSF-derived EVs is challenging due to the paucity of material for study. Whilst variability of quantification is rarely reported in mass spectrometry of EVs, one study of cell culture-derived EVs demonstrated that variability was strongly related to peptide load, with median coefficient of variation at 25.2% for ultra-low 2 ng peptide quantities using a 15-minute gradient, falling to 4.7% with 200 ng at 44-minute gradient [58]. However, variability is typically much higher in biofluid samples such as blood and CSF, including in EVs [59]. Unexpectedly, we found that immunocapture from the largest starting CSF volume showed the highest variability, indicating that it cannot necessarily be reduced by increasing starting material. In our experiments the CSF volumes were normalised to 1 mL for immunocapture, so the dilution of CSF for 200 and 500 µL may have aided more reproducible capture. Bound EVs were washed with a standard low percentage of Tween-20 detergent (0.05%) to reduce non-specific binding, but evidence suggests that EV are resistant to up to 5% Tween-20, so higher concentrations could be used to reduce co-isolation of soluble protein [60, 61]. Small protocol modifications, therefore, might have significant impacts on the purity of EVs and the resulting proteomic depth.

Our immunocapture protocol was highly robust, showing clear increases in captured material with increased CSF starting volume, indicating capture specificity. Due to the challenges of eluting intact EVs however, other orthogonal characterisation methods could not be carried out. CD81, CD9 and CD63 tetraspanins are abundant on EVs of many cellular origins, so it is unsurprising that the core EV proteome comprised predominantly blood-derived proteins (as is seen in whole CSF) and choroid plexus-derived proteins, in line with previous studies [10, 14]. With the identification of suitable cell type-specific or sub-cellular origin-specific membrane markers, however, it should be possible to apply the same methodology with different antibodies, nanobodies, or aptamers to effectively enrich for these populations. Whilst present in almost all samples, we did not find evidence to suggest that the most used neuronal EV marker, L1CAM, was present in a membrane-bound over soluble form, with its abundance lowest in immunocaptured samples and lacking cytoplasmic precursor peptide detection. However, the small size of the cytoplasmic region, and the high hydrophobicity of membrane-spanning regions may negatively impact the ability to detect such peptides, so this does not prove their absence. For an alternative target, ATP1A3 [34], only a single precursor peptide was detected in only three samples, but this was a cytoplasmic peptide, providing stronger evidence that the full-length protein is present primarily in a membrane-spanning form. Notably, ATP1A3, like tetraspanin proteins, is a multi-pass membrane protein, without extended extracellular regions that are susceptible to proteolytic cleavage. Therefore, multi-pass membrane proteins with neuronally-restricted expression may provide more robust targets for neuronal EV capture direct from CSF (enabling reduced volume requirements) if, and where, reagents targeting extracellularly exposed regions are available.

## Conclusions

In conclusion, our study demonstrates that immunocapture enables high-depth proteomic profiling of extracellular vesicles from lower starting volumes (200–500 µL CSF) than used for SEC, addressing a key limiting factor in the use of CSF EVs for biomarker discovery. The same methods could be applied to study other biological cargoes of interest in EVs from CSF and other biofluids, such as RNA, but will require additional validation for these applications [62–65]. Whilst our work makes use of tetraspanin antibodies for capture, the same methodology can be applied using antibodies for cell type-specific or sub-cellular origin-specific membrane markers.

## Supplementary Information

Supplementary Material 1

Supplementary Material 2

## Data Availability

We have submitted all relevant data of our experiments to the EV-TRACK knowledgebase (EV-TRACK ID: EV250021) [66]. The mass spectrometry data have been deposited to the ProteomeXchange Consortium ([http://proteomecentral.proteomexchange.org](http:/proteomecentral.proteomexchange.org)) via the PRIDE partner repository with the dataset identifier PXD061653.

## Abbreviations

CSF: Cerebrospinal fluid
CNS: Central nervous system
EV: Extracellular vesicle
EP: Extracellular particle
DIA: Data-independent-acquisition
NVEPs: Non-vesicular extracellular particles
HDL: High-density lipoprotein
LDL: Low-density lipoprotein
VLDL: Very- low-density lipoprotein
SEC: Size exclusion chromatography
IC: Immunocapture
MBR: Match between runs
