# Supplementary material for: Tetraspanin-based immunocapture for high-depth proteomic profiling of extracellular vesicles from cerebrospinal fluid for biomarker discovery
Source: Clin Proteomics. 2026 Jan 17;23:8. doi: 10.1186/s12014-025-09579-9 (PMC12895866; doi:10.1186/s12014-025-09579-9)
Supplement: Supplementary file 1 — Supplementary Material 1 [file 12014_2025_9579_MOESM1_ESM.pdf]

Tetraspanin-based immunocapture for high-depth proteomic profiling of extracellular vesicles from cerebrospinal fluid for biomarker discovery

Elizabeth R Dellar [1#], Iolanda Vendrell [2], Roman Fischer [2,3], Alexander G Thompson [1#.]

Affiliations:

1. Nuffield Department of Clinical Neurosciences, University of Oxford.

2. Target Discovery Institute, Centre for Medicines Discovery, Nuffield Department of Medicine, University of Oxford .

3. Chinese Academy of Medical Science Oxford Institute, Nuffield Department of Medicine, University of Oxford.

Subject	Diagnosis	Age at LP	Sex
1	Transverse myelitis	57	F
2	Idiopathic intracranial hypertension	35	F
3	Multiple sclerosis	55	F
4	Idiopathic intracranial hypertension	22	F
5	Transient limb symptoms with no final diagnosis	39	M
6	Optic neuritis	49	F
7	Idiopathic intracranial hypertension	43	F
8	Idiopathic intracranial hypertension	32	M
9	Idiopathic intracranial hypertension	27	F
10	Idiopathic intracranial hypertension	47	F

Supplementary Table 1

Demographic details of subjects used to produce CSF pool. LP; lumbar puncture.

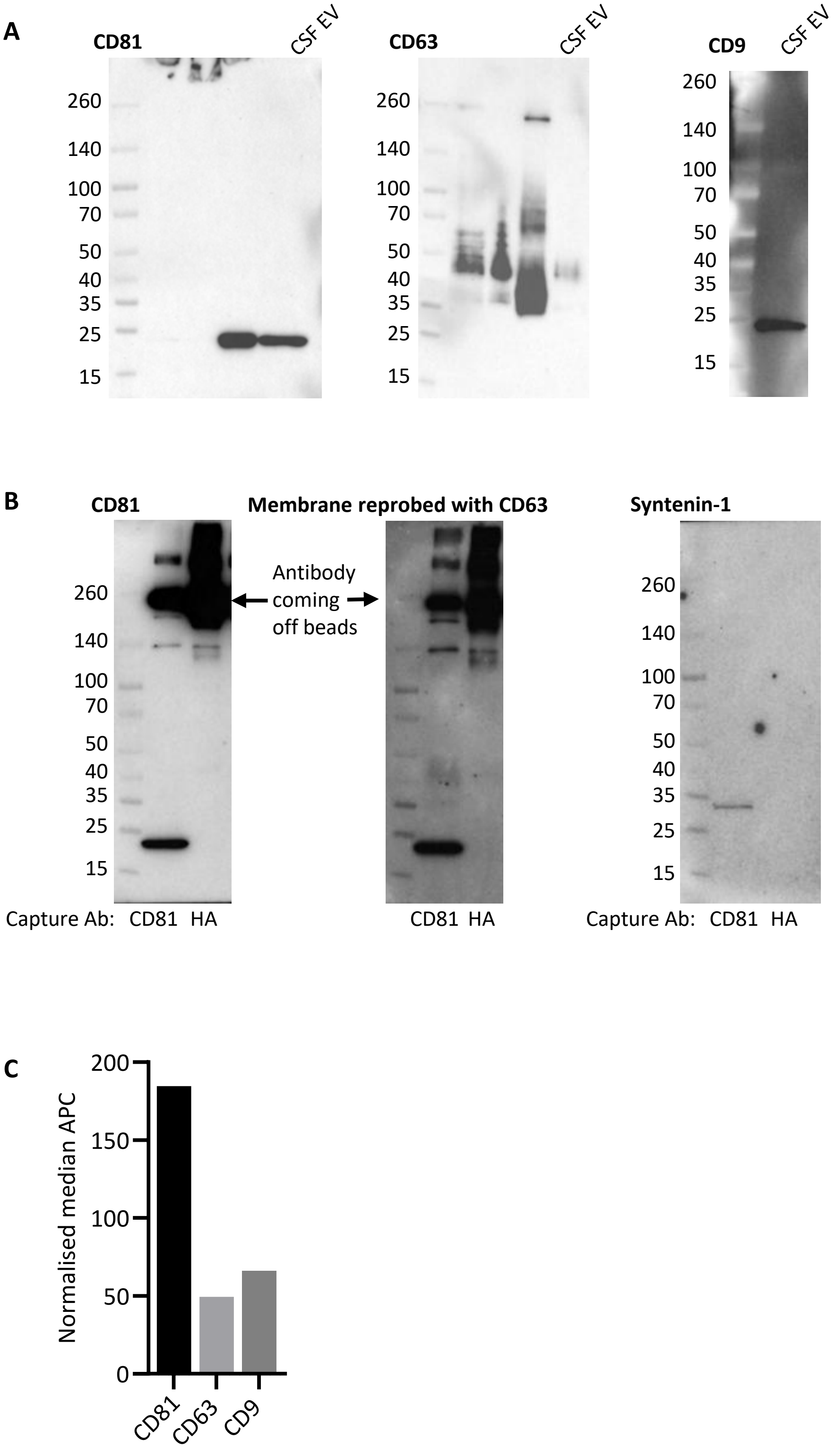

Supplementary Figure 1

(A) Full blots for Figure 1B.
(B) Full blots for Figure 1C.
(C) Tetraspanin abundance for whole CSF from MACSPlex EV flow cytometry assay (n=1).

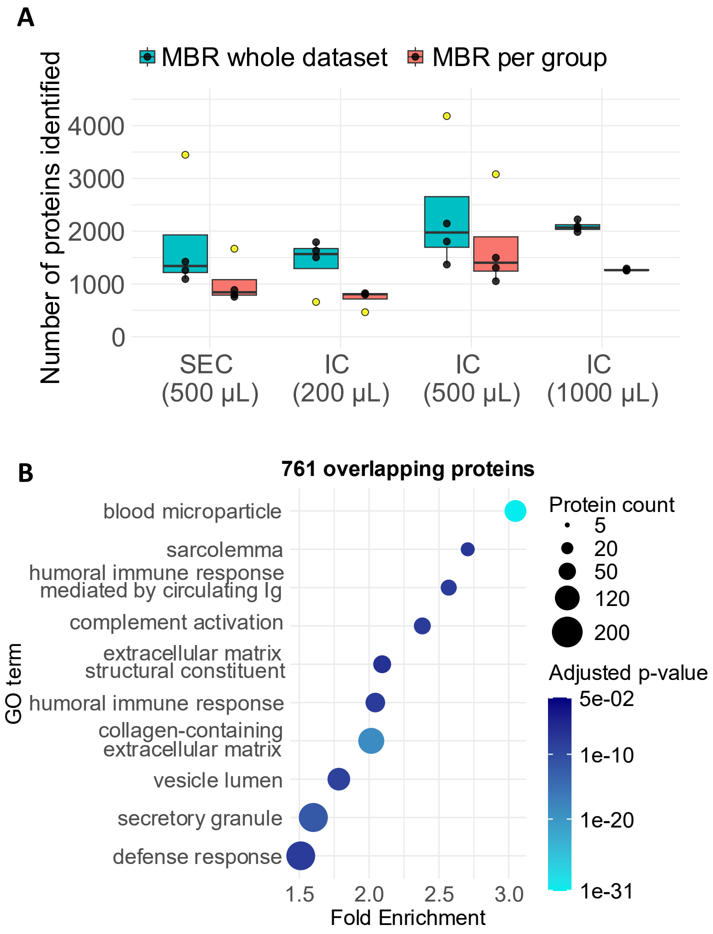

Supplementary Figure 2

(A) Total number of proteins identified in mass spectrometry, with outlying samples highlighted in yellow. (B) Overrepresentation analysis of proteins detected in EVs enriched by either SEC or IC from 500 µL starting CSF volume, showing top ten most significant terms. IC, immunocapture; SEC, size-exclusion chromatography.

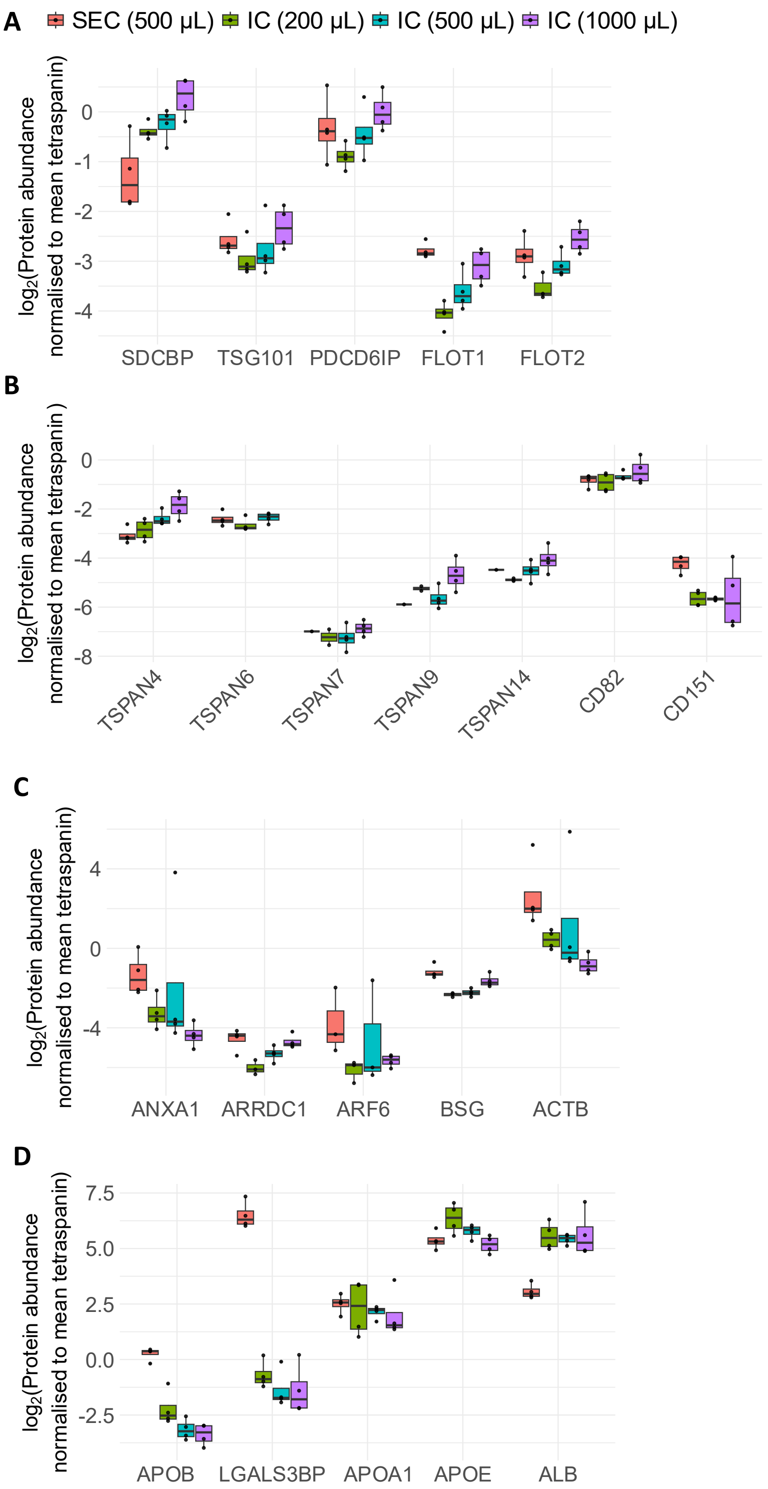

Supplementary Figure 3

(A) Abundance of typical markers associated with endosomal-derived EVs after normalisation to average abundance of CD81, CD63 and CD9. (B) Abundance of all non-canonical tetraspanin EV markers, (C) markers typically associated with plasma membrane-shed EVs and (D) common co-isolates in CSF and other biofluids.

IC, immunocapture; SEC, size-exclusion chromatography.

Sample	Protein Type																	
	Membrane (Single-pass)	Membrane (Multi-pass)	Membrane (Peripheral)	Membrane (Lipid-anchor)	Secreted	Other	Secreted, Membrane (Lipid-anchor), Membrane (Peripheral)	Membrane (Single-pass), Secreted	Membrane (Multi-pass), Secreted	Secreted, Membrane (Peripheral)	Membrane (Lipid-anchor), Membrane (Peripheral)	Secreted, Membrane (Lipid-anchor)	Membrane (Single-pass), Secreted, Membrane (Lipid-anchor)	Membrane (Single-pass), Secreted, Membrane (Peripheral)	Membrane (Single-pass), Membrane (Lipid- anchor)	Membrane (Multi-pass), Membrane (Peripheral)	Membrane (Single-pass), Membrane (Peripheral)	Membrane (Single-pass), Membrane (Multi-pass)
GroupMBR_200_1	271	121	94	87	448	634	1	64	5	19	5	11	2	1	2	1	1	1
GroupMBR_200_2	227	94	82	73	384	532	0	54	5	16	4	9	2	1	1	0	1	1
GroupMBR_200_4	228	127	95	76	389	603	0	55	3	14	5	8	2	1	2	2	1	1
GroupMBR_500_1	261	139	105	103	407	657	0	65	5	15	6	10	2	1	4	0	1	1
GroupMBR_500_3	311	184	134	112	455	797	0	74	5	21	7	11	2	1	4	1	1	1
GroupMBR_500_4	214	98	76	82	367	422	0	50	5	12	5	8	2	1	3	0	1	1
GroupMBR_1000_1	314	171	120	114	418	761	0	77	6	16	7	14	2	1	6	0	1	0
GroupMBR_1000_2	307	174	138	117	417	784	0	78	5	15	7	13	2	1	5	1	1	0
GroupMBR_1000_3	352	183	131	119	466	803	1	82	6	18	7	16	2	1	7	1	1	1
GroupMBR_1000_4	304	166	123	111	430	700	1	75	6	14	7	12	2	1	5	0	1	0
GroupMBR_SEC_1	153	95	74	79	327	437	0	42	2	13	5	7	2	1	2	0	1	0
GroupMBR_SEC_2	166	100	91	84	343	535	0	49	2	12	5	8	2	1	3	0	1	0
GroupMBR_SEC_3	122	78	62	66	292	386	0	37	2	12	4	6	2	1	1	0	1	0

Supplementary Table 2: Number of proteins of each type detected within dataset

Data from match between runs applied on the dataset as a whole, three outlying samples excluded based on total number of proteins detected. Shaded area shows categories used in Figure 3.

Sample	Protein Type																	
	Membrane (Single-pass)	Membrane (Multi-pass)	Membrane (Peripheral)	Membrane (Lipid-anchor)	Secreted	Other	Secreted, Membrane (Lipid-anchor), Membrane (Peripheral)	Membrane (Single-pass), Secreted	Membrane (Multi-pass), Secreted	Secreted, Membrane (Peripheral)	Membrane (Lipid-anchor), Membrane (Peripheral)	Secreted, Membrane (Lipid-anchor)	Membrane (Single-pass), Secreted, Membrane (Lipid-anchor)	Membrane (Single-pass), Secreted, Membrane (Peripheral)	Membrane (Single-pass), Membrane (Lipid- anchor)	Membrane (Multi-pass), Membrane (Peripheral)	Membrane (Single-pass), Membrane (Peripheral)	Membrane (Single-pass), Membrane (Multi-pass)
GroupMBR_200_1	15.3	6.8	5.3	4.9	25.3	35.9	0.1	3.6	0.3	1.1	0.3	0.6	0.1	0.1	0.1	0.1	0.1	0.1
GroupMBR_200_2	15.3	6.3	5.5	4.9	25.8	35.8	0.0	3.6	0.3	1.1	0.3	0.6	0.1	0.1	0.1	0.0	0.1	0.1
GroupMBR_200_4	14.1	7.9	5.9	4.7	24.1	37.4	0.0	3.4	0.2	0.9	0.3	0.5	0.1	0.1	0.1	0.1	0.1	0.1
GroupMBR_500_1	14.6	7.8	5.9	5.8	22.8	36.9	0.0	3.6	0.3	0.8	0.3	0.6	0.1	0.1	0.2	0.0	0.1	0.1
GroupMBR_500_3	14.7	8.7	6.3	5.3	21.5	37.6	0.0	3.5	0.2	1.0	0.3	0.5	0.1	0.0	0.2	0.0	0.0	0.0
GroupMBR_500_4	15.9	7.3	5.6	6.1	27.2	31.3	0.0	3.7	0.4	0.9	0.4	0.6	0.1	0.1	0.2	0.0	0.1	0.1
GroupMBR_1000_1	15.5	8.4	5.9	5.6	20.6	37.5	0.0	3.8	0.3	0.8	0.3	0.7	0.1	0.0	0.3	0.0	0.0	0.0
GroupMBR_1000_2	14.9	8.4	6.7	5.7	20.2	38.0	0.0	3.8	0.2	0.7	0.3	0.6	0.1	0.0	0.2	0.0	0.0	0.0
GroupMBR_1000_3	16.0	8.3	6.0	5.4	21.2	36.5	0.0	3.7	0.3	0.8	0.3	0.7	0.1	0.0	0.3	0.0	0.0	0.0
GroupMBR_1000_4	15.5	8.5	6.3	5.7	22.0	35.8	0.1	3.8	0.3	0.7	0.4	0.6	0.1	0.1	0.3	0.0	0.1	0.0
GroupMBR_SEC_1	12.3	7.7	6.0	6.4	26.4	35.2	0.0	3.4	0.2	1.0	0.4	0.6	0.2	0.1	0.2	0.0	0.1	0.0
GroupMBR_SEC_2	11.8	7.1	6.5	6.0	24.5	38.2	0.0	3.5	0.1	0.9	0.4	0.6	0.1	0.1	0.2	0.0	0.1	0.0
GroupMBR_SEC_3	11.4	7.3	5.8	6.2	27.2	36.0	0.0	3.5	0.2	1.1	0.4	0.6	0.2	0.1	0.1	0.0	0.1	0.0

Supplementary Table 3: Percentage of proteins of each type detected within each sample

Data from match between runs applied on the dataset as a whole, three outlying samples excluded based on total number of proteins detected. Shaded area shows categories used in Figure 3.

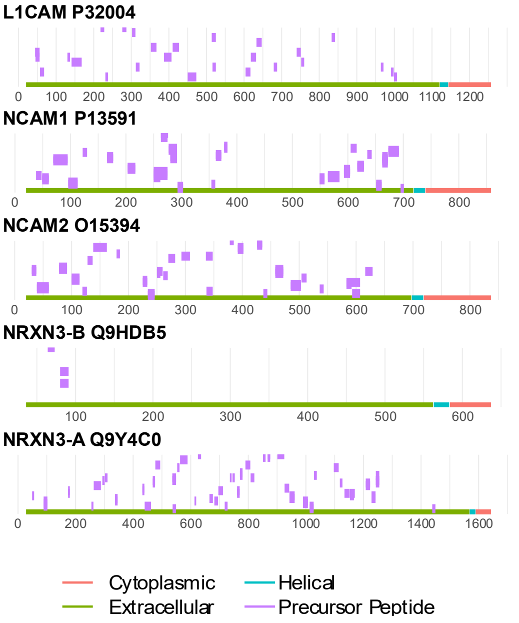

Supplementary Figure 4

Mapping of precursor peptides for literature-identified neuronal EV immunocapture targets from mass spectrometry against canonical full-length sequence and topological data (cytoplasmic, extracellular and helical transmembrane domains) from Uniprot for proteins in whole CSF data from Dellar *et al* (2024). ATP1A3 and GRIA2 were not detected.
